# Modest and variable efficacy of pre-exposure hydroxocobalamin and dicobalt edetate in a porcine model of acute cyanide salt poisoning

**DOI:** 10.1080/15563650.2019.1628969

**Published:** 2019-08-07

**Authors:** Adrian Thompson, Michael Dunn, Robert D Jefferson, Kosala Dissanayake, Frances Reed, Rachael Gregson, Stephen Greenhalgh, R Eddie Clutton, Peter G Blain, Simon HL Thomas, Michael Eddleston

**Affiliations:** a Department of Pharmacology, Toxicology, & Therapeutics, University/BHF Centre for Cardiovascular Science, University of Edinburgh, Edinburgh, UK;; b Medical Toxicology Centre, University of Newcastle, Newcastle upon Tyne, UK;; c Wellcome Critical Care Laboratory for Large Animals, Roslin Institute, Royal (Dick) School of Veterinary Studies, University of Edinburgh, Edinburgh, UK

**Keywords:** Cyanide, antidotes, efficacy

## Abstract

**Background:** Dicobalt edetate and hydroxocobalamin are widely used to treat hydrogen cyanide poisoning. However, comparative and quantitative efficacy data are lacking. Although post-exposure treatment is typical, it may be possible to administer these antidotes before exposure to first attenders entering a known site of cyanide release, as supplementary protection to their personal protective equipment.

**Methods:** We established an anaesthetised Gottingen minipig model of lethal bolus potassium cyanide (KCN) injection to simulate high dose hydrogen cyanide inhalation. Doses were similar to human lethal doses of KCN. Dicobalt edetate and hydroxocobalamin were administered shortly before KCN and their effect on metabolic and cardiovascular variables and survival time were measured.

**Results:** Increases in arterial lactate were similar after 0.08 and 0.12 mmol/kg KCN. KCN 0.08 mmol/kg was survived by 4/4 animals with moderate cardiovascular effects, while the 0.12 mmol/kg dose was lethal in 4/4 animals, with a mean time to euthanasia of 28.3 (SEM: 13.9) min. Administration of dicobalt edetate (0.021 mmol/kg, 8.6 mg/kg) or hydroxocobalamin (0.054 mmol/kg, 75 mg/kg) at clinically licenced doses had modest effect on lactate concentrations but increased survival after administration of KCN 0.12 mmol/kg (survival: dicobalt edetate 4/4, hydroxocobalamin 2/4) but not 0.15 mmol/kg (0/4 and 0/4, respectively). In a subsequent larger study, doubling the dose of hydroxocobalamin (0.108 mmol/kg, 150 mg/kg) was associated with a modest but inconsistent increased survival after 0.15 mmol/kg KCN (survival: control 0/8, 75 mg/kg 1/10, 150 mg/kg 3/10) likely due to variable pharmacokinetics.

**Conclusions:** In this porcine study of cyanide exposure, with pre-exposure antidote administration, licenced doses of dicobalt edetate and hydroxocobalamin were effective at just lethal doses but ineffective at less than twice the estimated LD_50_. The efficacy of a rapidly-administered double-dose of hydroxocobalamin was limited by variable pharmacokinetics. In clinical poisoning scenarios, with delayed administration, the antidotes are likely to be even less effective. New antidotes are required for treatment of cyanide exposures appreciably above the minimum lethal dose.

## Introduction

Hydrogen cyanide and cyanide salts are highly toxic industrial chemicals that may be used in terrorist attacks [1–3]. Dicobalt edetate and hydroxocobalamin are two established and widely used antidotes [4–10]. Dicobalt edetate is the licenced UK antidote and has been used since Paulet’s work on cyanide antidotes in the 1950s [11]. Hydroxocobalamin has been introduced more recently [6]. Few data are available on their comparative efficacy and no randomised controlled trials have been performed in humans. Analysis of their binding capacity suggests that they bind only modest quantities of cyanide (Box 1); however, it is possible that small reductions in free cyanide concentration may be clinically beneficial.

Dicobalt edetate is associated with hypertension, tachycardia, and retrosternal pain [10], while hydroxocobalamin is associated with few adverse effects [7]. It may, therefore, be possible to rapidly administer hydroxocobalamin to hazardous area response teams (HART) first attenders before they enter a site after known cyanide release, as a back-up for their personal protective equipment.

However, a little-discussed problem with hydroxocobalamin is its marked pharmacokinetic (PK) variation observed after intravenous (IV) administration. In a dog study (N106342) used for licencing, the C_max_ could not be calculated due to the high degree of variation [12] (Table, p15). In a porcine study of IV administration [13], C_max_ varied 2.7-fold between animals (Eddleston et al. unpublished). Marked variation has also been seen in human PK studies, with C_max_ varying from 359 to 1360 mg/L (mean: 813 mg/L) in one study of nine individuals [14] and from 288 to 995 mg/L in a licencing study reported to the European Medicines Agency [12]. The clinical consequences of this PK variation are not yet clear.

Box 1 Chelation capacity of the two antidotes.

- The initial dose of hydroxocobalamin HCl (molecular weight = 1382) used in this study was 75 mg/kg, or 0.054 mmol/kg. Hydroxocobalamin binds cyanide in a 1:1 molar ratio.
- Dicobalt edetate administered at 8.6 mg/kg equates to 0.021 mmol/kg (assuming that the given weight in the formulated product refers to the anhydrous formula weight of 406). Dicobalt edetate binds cyanide in a 1:2 molar ratio under physiological conditions [45].
- If the administered quantities were fully available to react with cyanide, a maximum of 0.054 mmol/kg of KCN could be detoxified by hydroxocobalamin and 0.042 mmol/kg by dicobalt edetate. Double the dose of hydroxocobalamin would detoxify 0.108 mmol/kg.
- In the single bolus dose model reported here, the minimum lethal dose for KCN was estimated to be between 0.08 mmol/kg (100% survival to 90 min) and 0.12 mmol/kg (100% lethal at 90 min).
- The theoretical maximum cyanide binding capacity of the recommended human doses of these antidotes is approximately half of the minimum lethal dose for the animals in this model. The double hydroxocobalamin dose is approximately equal to the minimum lethal dose.

Trials to assess the efficacy of antidotes for cyanide exposure cannot be done in humans; studies have therefore been done in animals including rodents [15], rabbits [16], dogs [17], and pigs [18,19]. Porcine models of KCN poisoning are probably most clinically relevant because pigs are relatively close to humans in terms of physiology and metabolism [20] and are widely used for toxicology studies [21].

Published large animal antidote studies have commonly involved infusion of cyanide salts until a pre-defined endpoint (such as a 50% reduction in mean arterial pressure (MAP), apnoea or cardiac arrest) was reached [18,22–24]. At this point, the infusion was stopped, an antidote administered, and effects on cardiorespiratory responses compared. While these models are useful for proof of concept, they are sensitive and able to detect very small improvements in efficacy because the dose of cyanide received approximates the minimum dose likely to be fatal. A small antidotal effect may be protective at this dose, while providing little efficacy for higher doses. The models do not assess treatment of toxicity from an acute (bolus) exposure, as will occur in an attack, or compare the efficacy between antidotes at different estimated doses.

We established a pig model of KCN poisoning that could be used to compare the efficacy of the two antidotes. We used bolus intravenous doses of KCN to more closely simulate a sudden highly toxic exposure by inhalation. We gave the antidotes immediately before exposure to maximise the antidotes’ plasma concentrations at the time of poisoning and test the possibility for prophylactic use. The KCN doses used (starting at 5.25 mg/kg) were modestly higher than the estimated lethal dose in humans (150–300 mg; 2–4 mg/kg in a 75 kg adult) [25], perhaps due to species differences or the use of ventilation in the model. By doing so, we aimed to establish the maximum protective benefit that these antidotes can give at the doses chosen.

## Methods

### Animals

Experiments involved male Göttingen minipigs (*n* = 64, Ellegaard Minipigs ApS, Dalmose, Denmark) with mean weight 13.7 (SEM: 0.2) kg. Animals were barrier bred, shown to be free of infections before shipment, and treated in accordance with the Animals (Scientific Procedures) Act of 1986. The study was performed under Home Office Licence after institutional ethics review.

### Cyanide and drugs

All compounds were obtained from Sigma-Aldrich, UK, unless otherwise indicated. KCN and NaCN solutions were prepared in phosphate buffered saline (pH: 7.4–7.5) 1–2 days before the study and stored in stoppered syringes at 4 °C. Injection volumes in the range 5–9 mL were adjusted to give the required dose (mmol/kg) based on the animal’s weight on the day of the experiment. KCl (Martindale Pharmaceuticals, UK) 15% (w/v) solution was diluted with normal saline to give the required dose in 10 mL.

Hydroxocobalamin hydrochloride was dissolved in normal saline to produce a 33.3 mg/mL solution immediately before use. Dicobalt edetate (Alliance Pharmaceuticals, UK) was obtained as a 15 mg/mL injectable solution. Volumes were calculated to give the required doses based on the animal’s weight on the experiment day.

### Study design

Drug-naive animals were kept in pens and acclimatised under the care of institutional veterinary surgeons before the study as per the study laboratory protocol. Animals were fed a standard diet but fasted overnight preceding the study; water was available freely. Animals were weighed on the morning of the study.

The experiment was conducted in phases, with the first phase being used to identify approximate maximum survivable and minimal lethal (MLD) doses of KCN that required poisoned pigs to be euthanized within a target time of 90 min. Based on the KCN dose used to cause cardiac arrest in a previous pig study of hydroxocobalamin [23] and our own pilot studies (unpublished), doses of 5.25 mg/kg (0.08 mmol/kg) and 7.5 mg/kg (0.12 mmol/kg) of KCN were selected for initial evaluation as a survivable dose and approximate MLD, respectively. These doses were significantly higher than the KCN doses used to induce apnoea in pig studies (1–2 mg/kg) [22,23]. In subsequent phases, antidotes were administered immediately before cyanide at the MLD and then against a 33% higher cyanide dose 10 mg/kg KCN (0.15 mmol/kg). Before the highest dose was evaluated, we investigated whether the potassium in the KCN could itself be causing a toxic effect. Equimolar KCl and NaCN were administered in the same manner as KCN, and the responses compared over a shorter 30 min period.

To reduce bias, animals were allocated to treatment groups using a random number list; allocation could not be predicted before randomisation.

### Anaesthesia and instrumentation

The first study began each day at around 08:00 and up to four studies were performed in one day. The methods of anaesthesia induction, central vascular access, and instrumentation together with continual cardiovascular monitoring of animals have been described previously [13,26]. Preanesthetic medication was intramuscular midazolam (0.5 mg/kg) and ketamine (5 mg/kg). When profoundly sedated, the animals were anaesthetised with 5% isoflurane in oxygen delivered by facemask and intubated by direct laryngoscopy after local anaesthesia to the hypopharynx with lidocaine 2% by a mucosal atomiser device. Anaesthesia was maintained to a clinically acceptable depth with isoflurane in oxygen delivered by a circle breathing system.

### Experimental protocol

#### Administration of KCN

After a 30 min stabilisation period, KCN solution was given by bolus injection over 40 s via a central venous line. The volume of KCN solution administered was always 0.5 mL/kg; for example, the concentration for the starting dose of 5.25 mg/kg (0.08 mmol/kg) was 10.5 mg/mL (0.16 mmol/mL) of KCN. This concentration was increased for higher doses to ensure that the same volume was given in all studies.

#### Evaluation of antidotes

Antidotes were administered shortly before the KCN challenge at *t* = 0 min. Hydroxocobalamin was given from -10 min until 0 min; dicobalt edetate was administered from -10 min until -5 min (followed by 1 mL/kg of 50% glucose from -5 min to 0 min as recommended by the manufacturer [27]). The UK licenced dose of hydroxocobalamin is 5 g (71.4 mg/kg in a 70 kg adult) over 15 min, repeated as necessary over 15–20 min [28]; we administered 75 mg/kg or 150 mg/kg of the hydrochloride salt by infusion over 10 min. The UK licenced dose of dicobalt edetate is 300 mg over 1 min (4.3 mg/kg in a 70 kg adult) followed immediately by 50 mL of 50% intravenous glucose solution, repeated as necessary within 5 min [28]. Because two doses could be given rapidly over a few minutes, we combined both doses and administered 8.6 mg/kg as an infusion over 5 min. In the final study, comparing hydroxocobalamin 75 mg/kg and 150 mg/kg, the antidote was given earlier, from -30 to -20 min to ensure effective distribution to the tissue before administration of cyanide.

#### Administration of NaCN and KCl

To determine whether potassium from the KCN solution contributed to the toxicity seen with the 0.15 mmol/kg KCN challenge, equimolar quantities of KCl (0.5 mL/kg of a 23 mg/mL solution) and NaCN (0.5 mL/kg of a 15 mg/mL solution) were each administered to groups of four pigs.

#### Blood sampling

Blood samples were taken at -30 and -10 min prior to administration of cyanide or KCl, then at 5 min and 10 min post challenge. Sampling continued at 10 min intervals for up to 90 min or until euthanasia. A 0.2 mL sample taken from the arterial catheter was used immediately for blood gas and lactate analyses on an EPOC blood analyser (Woodley Equipment Company Ltd, UK). It was not possible to measure arterial oxygen tensions in groups receiving high dose cyanide salts because of an interaction with the blood analyser. At the same time, 5 mL of blood was taken from the central venous catheter; 2 mL was frozen at -20 °C as whole blood for subsequent cyanide assay. The remaining sample was transferred to an EDTA-containing tube, centrifuged for 7 min at 3900 rpm, and the plasma component stored frozen.

#### Euthanasia

The animals were given a fatal overdose of IV sodium pentobarbital at the end of the study whilst under isoflurane anaesthesia or when there was cardiovascular decompensation that did not show improvement in less than 10 min towards a MAP of >45 mmHg.

### Assays

Detailed methodology is given in the Supplementary Appendix. Briefly, plasma hydroxocobalamin and cyanocobalamin concentrations were analysed using an HPLC method based on the work of Astier and Baud [29], and Chassaigne and Lobinski [30]. Cyanide was measured in whole blood using liquid chromatography-electrospray ionisation mass spectrometry (LC-ESI-MS) based on the work of Tracqui [31] and Lacroix [32]. Dicobalt edetate was measured as total cobalt in whole blood using inductively coupled plasma-mass spectrometry (ICP-MS) with collision cell technology and 7.5% (v/v) hydrogen in helium as the collision gas [33].

### Outcomes

The main study outcome for all but the last study was arterial blood lactate concentrations. Secondary outcomes included cardiovascular effects, time to euthanasia, and case fatality. Case fatality was the primary outcome for the final study.

### Statistical analysis

In the absence of previous data from this model, we did not perform a power calculation for the initial studies and used four animals in each group. However, to compare the hydroxocobalamin high dose against a lower dose and placebo, using mortality as an outcome, a power calculation indicated that group of 10 animals would allow detection of a treatment difference of 100% mortality in the control group and 30% mortality in a treatment group with 95% confidence and 80% power.

Primary data analysis was done in Prism 7 (GraphPad, San Diego, CA). All animals were included in the analysis. Pig weights plus clinical and biochemical outcomes were summarised with mean and SEM. Comparisons between groups were performed using *t*-tests. Statistical significance was assessed at *p* < .05. All *p* values represent 2-tailed calculations.

### Role of the funding source

The funders had no role in study design, data collection, data analysis, data interpretation or writing of the report. All authors had full access to all the study data and agreed with the decision to submit for publication.

## Results

### Identification of approximate maximum survivable and minimal lethal KCN doses

**Figure 1.**
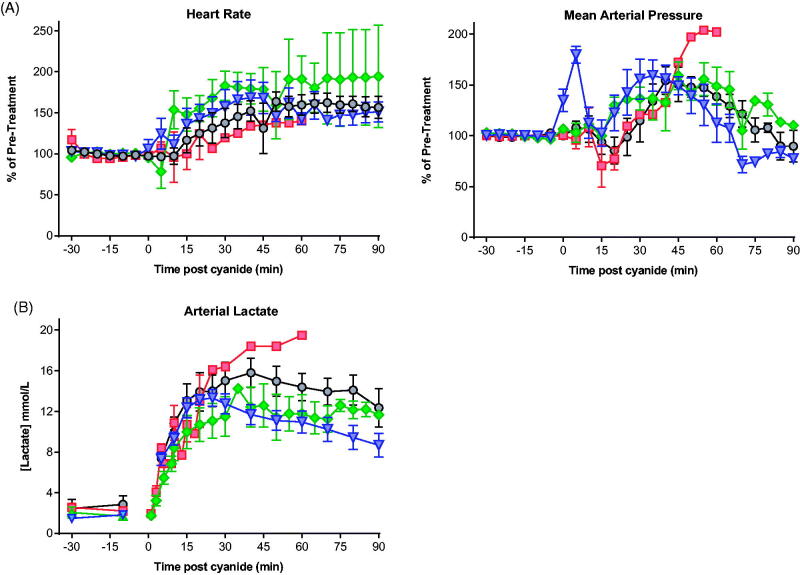
Cardiovascular and metabolic responses to cyanide with or without antidotes. (A) Cardiovascular (heart rate, MAP; 5 min intervals) and (B) metabolic (arterial lactate) responses to 0.08 mmol/kg KCN (circle) and 0.12 mmol/kg KCN (square) and to 0.12 mmol/kg KCN following pre-treatment with hydroxocobalamin (diamond) or dicobalt edetate (triangle). Values are mean ± SEM (for cardiovascular variables, of normalised responses [mean of -30 min and -10 min pre-cyanide exposure values = 100%]).

All four animals administered 0.08 mmol/kg KCN survived to the planned study duration of 90 min. Three of four showed an initial fall in MAP and pulse; all then showed hypertension and tachycardia (Figure 1(A)). These effects settled over about 70 min.

Arterial lactate peaked at a mean of 15.8 (SEM: 1.4) mmol/L at 40 min compared to a baseline of 2.7 (SEM: 0.58) mmol/L; *p* < .001; Figure 1(B)). It then fell modestly to a mean of 13.3 (SEM: 1.6) mmol/L by 90 min (*p* > .05 compared to 40 min).

**Figure 2.**
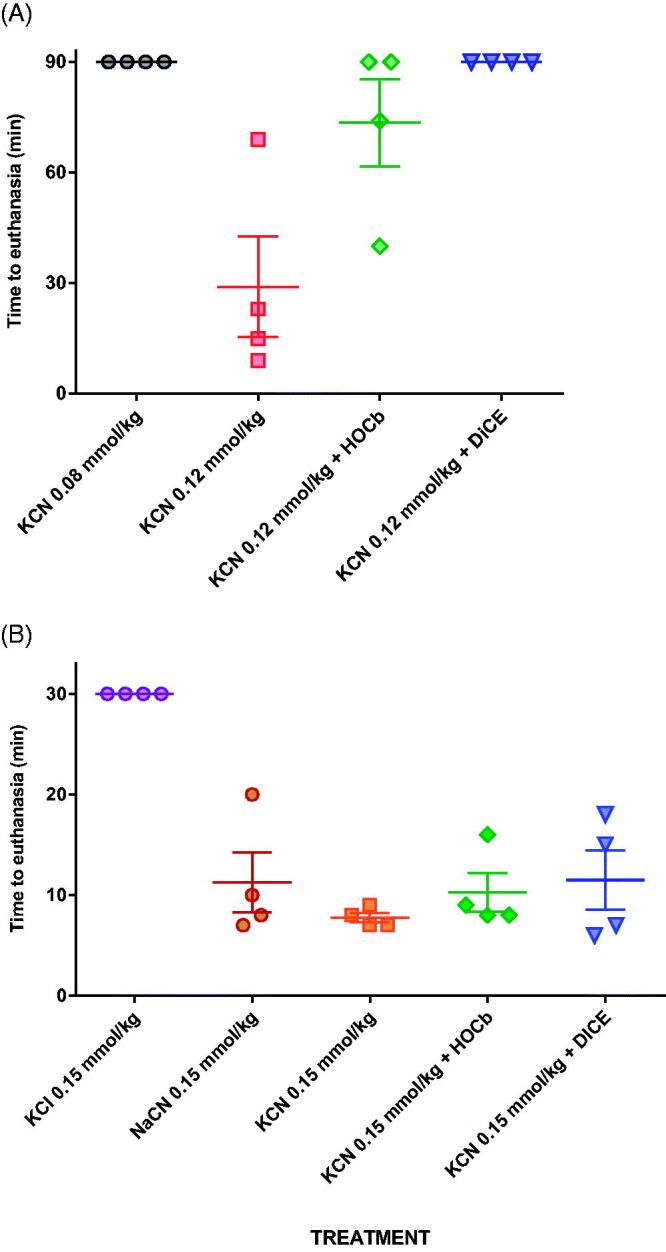
Survival post cyanide dosing. (A) Survival following exposure to 0.08 or to 0.12 mmol/kg KCN with or without antidotes. (B) Survival following exposure to 0.15 mmol/kg KCN, with or without antidotes, or equimolar KCl or NaCN. The studies had planned maximum durations of (A) 90 min and (B) 30 min. All animals that underwent euthanasia at this time had good cardiovascular function and were judged to have survived the procedure. Mean (SEM) time to euthanasia are shown for each group.

All four animals that received 0.12 mmol/kg KCN required euthanasia due to sudden cardiac decompensation (Figure 1(A)) before 90 min (mean survival time: 28.3 min; Figure 2(A)). Arterial lactate concentrations rose at a similar rate to that following the 0.08 mmol/kg dose (maximum: 19.5 mmol/L at 60 min in the one animal that survived to this time; Figure 1(B)). Measured peak blood cyanide (CN^−^, molecular weight 26) concentrations of 328.8 and 352.1 μM occurred at the first time point, 5 min after KCN administration, in groups treated with 0.08 and 0.12 mmol/kg KCN, respectively (Table 1).

**Table 1.** Free cyanide concentrations following administration of 0.08, 0.12 and 0.15 mmol/kg KCN, and 0.15 mmol/kg NaCN.

	Cyanide salt:	KCN							NaCN
	Dose (mmol/kg):	0.08	0.12			0.15			0.15
	Antidote:	None	None	HOCb	DiCE	None	HOCb	DiCE	None
Time (min)									
−30		0	0	0	0	0	0	0	0
5		328.8 (17.1)	352.1 (34.9)	ND	348.3 (37.8)	682.5 (50.6)	388.6 (20.3)*	509.1 (29.1)*	684.7 (66.2)
10		309.0 (53.0)	315.8 (13.0)	272.9 (27.6)	262.8 (21.0)				

Hydroxocobalamin (HOCb, 75 mg/kg) and dicobalt edetate (DiCE) were evaluated against 0.12 and 0.15 mmol/kg KCN challenges. Values are mean (SEM) blood cyanide concentrations (µmol/L CN^−^). Values marked with an asterisk are significantly different (*p* < .05) from the equimolar cyanide challenge without antidote. ND: not done.

These two KCN doses were then taken as estimates of the maximum survivable dose and MLD, as it was considered unnecessary and unethical to study further animals to refine further these estimates.

### Evaluation of antidotes against an approximate minimal lethal cyanide dose

Both antidotes showed efficacy when tested against 0.12 mmol/kg KCN. Four of four animals and 2/4 animals survived to 90 min after dicobalt edetate or hydroxocobalamin, respectively (Figure 2(A)). Two hydroxocobalamin treated animals survived to 31 min and 74 min post-KCN exposure. Despite improved survival, the antidotes only modestly reduced the peak arterial lactate concentration (hydroxocobalamin: 14.2 mmol/L at 35 min, dicobalt edetate: 13.3 mmol/L at 25 min; Figure 1(B)) compared to no antidote (19.5 mmol/L at 60 min) and had little effect on cardiovascular toxicity (Figure 1(A)).

**Figure 3.**
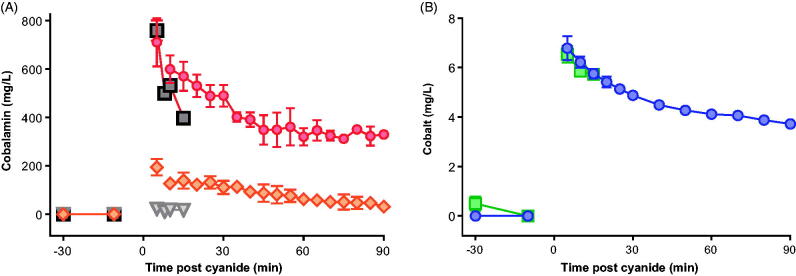
Pharmacokinetics of the antidotes. (A) Mean (SEM) blood hydroxocobalamin (orange diamond) and cyanocobalamin (red circle) concentrations after infusion of hydroxocobalamin from -10 to 0 min relative to a 0.12 mmol/kg KCN challenge. The concentrations of hydroxocobalamin (triangle) and cyanocobalamin (square) are also shown at baseline and at 5 min after 0.15 mmol/kg KCN. (B) Mean (SEM) blood cobalt concentration (circle) after infusion of dicobalt edetate from -10 to -5 min relative to a 0.12 mmol/kg KCN challenge. The cobalt concentration is also shown at baseline and at 5 min after 0.15 mmol/kg KCN ( square). Only a 5 min sample was obtained following the higher KCN challenge due to all animals requiring euthanasia 6–9 min post challenge.

Neither antidote reduced peak cyanide concentrations following the 0.12 mmol/kg challenge (Table 1). Mean peak measured total cobalamin concentration (904 mg/L) occurred 5 min post-KCN exposure in the group given 0.12 mmol/kg of KCN (Figure 3(A)). Most of the cobalamin was in the cyanocobalamin form at 5 min (79%) indicating that the rate of combination of hydroxocobalamin with cyanide (and conversion to cyanocobalamin) was unlikely to have limited its efficacy. There was 2.4-fold variation in measured C_max_ at 5 min for hydroxocobalamin between animals and 1.9-fold variation in C_max_ for cyanocobalamin.

The dicobalt edetate (measured as cobalt) concentration was 6.8 mg/L in animals 5 min after administration of 0.12 mmol/kg KCN (Figure 3(B)). The mean cobalt half-life was 157.5 (SEM: 17.1) min, indicating that clearance should not greatly reduce the efficacy of dicobalt edetate in this experiment.

### Exclusion of potassium toxicity

Before proceeding to evaluate the antidotes against a higher KCN dose (0.15 mmol/kg), we examined whether toxicity was due in part to potassium, since potassium toxicity would reduce apparent cyanide antidote efficacy.

**Figure 4.**
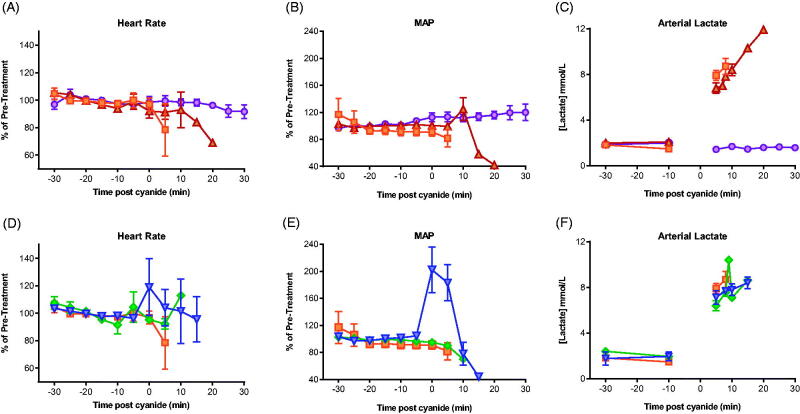
Effect of cyanide and potassium on cardiovascular function and lactate. (A–C) Cardiovascular (A: heart rate; B: MAP) and metabolic (C: arterial lactate) responses to 0.15 mmol/kg KCN (orange square) or to equimolar quantities of KCl ( circle) or NaCN (upwards triangle). (D–F) Cardiovascular (D: heart rate; E: MAP) and metabolic (F: arterial lactate) responses to 0.15 mmol/kg KCN alone (square) or after hydroxocobalamin (diamond) or dicobalt edetate (downwards triangle) antidotes. Values are means ± SEM (for cardiovascular variables, of normalised responses [mean of -30 min and -10 min pre-cyanide exposure values = 100%]).

Equimolar (0.15 mmol/kg) KCl and NaCN were given by IV administration over 40 s. KCl had no effect on cardiovascular function or arterial lactate concentration (1.6 mmol/L at 30 min compared to 1.9 mmol/L pre-treatment, *p* = .185) (Figure 4(A–C)). All animals were well at the time of euthanasia, 30 min post exposure (Figure 2(B)). In contrast, animals treated with 0.15 mmol/kg of KCN or NaCN showed similar rapid changes in cardiovascular function associated with rising lactate concentrations (Figure 4(A–C)). After 5 min, mean lactate concentrations had risen significantly to 7.9 [SEM: 0.44] mmol/L with KCN and 6.8 [SEM: 0.45] mmol/l with NaCN [both *p* < .001 compared to pre-exposure]; there was no difference between CN groups, *p* = .130).

The measured peak blood cyanide concentrations at 5 min were 682.5 and 684.7 mol/L in the groups receiving 0.15 mmol/kg KCN and NaCN. Mean times of euthanasia were similar between groups: 7 min for KCN and 8 min for NaCN (*p* = .33, Figure 2(B)). These results indicate that the toxicity seen with KCN in this model is due to cyanide and not potassium.

### Evaluation of antidotes against the higher cyanide dose

Treatment with either hydroxocobalamin or dicobalt edetate had no protective effect against the toxicity of 0.15 mmol/kg KCN. All animals showed a rapid rise in arterial lactate concentrations (Figure 4(F)) at 5 min post CN exposure to 7.9 (SEM: 0.44) mmol/L (controls), 6.4 (0.43) mmol/L (hydroxocobalamin), and 7.1 (0.60) mmol/L (dicobalt edetate; all *p* < .001 vs. pre-treatment values). The between-treatment differences at 5 min were not significant (*p* = .051 and 0.338, comparing controls with hydroxocobalamin and dicobalt edetate, respectively). MAP declined rapidly in all animals (Figure 4(E)), with similar mean survival times of around 7 min in control and treatment groups (Figure 2, *p* = .8 for comparisons of both treatment groups with control).

Compared to no antidote (peak mean cyanide concentration: 682.5 mol/L), both hydroxo-cobalamin and dicobalt edetate reduced cyanide concentrations at 5 min post exposure to 0.15 mmol/kg KCN (388.6 mol/L [*p* = .002 vs. control] and 509.1 mol/L [*p* = .022], respectively; Table 1). However, the observed cyanide concentrations were still higher than those seen after 0.12 mmol/kg KCN challenge and the antidotes did not prevent a rise in lactate concentrations (Figure 4(F)) or delay death (Figure 2(B)).

Peak total cobalamin concentration at 5 min post-KCN exposure was 780 mg/L; again, most of the cobalamin was in the cyanocobalamin form (97.0%, Figure 3(A)). A cobalt concentration of 6.6 mg/L was seen in the only sample (5 min) taken post-KCN exposure in the 0.15 mmol/kg treatment group (due to a rapid need for euthanasia).

### Evaluation of a double hydroxocobalamin dose

We finally tested in a larger randomised controlled study (n = 10 for antidote groups) whether rapid administration of a double dose of hydroxocobalamin (150 mg/kg, 10.5 g for a 70 kg adult) given over 10 min (from *t*= −30 min to *t*= −20 min before cyanide) would substantially improve efficacy compared to a standard 75 mg/kg (5 g) dose or placebo.

**Figure 5.**
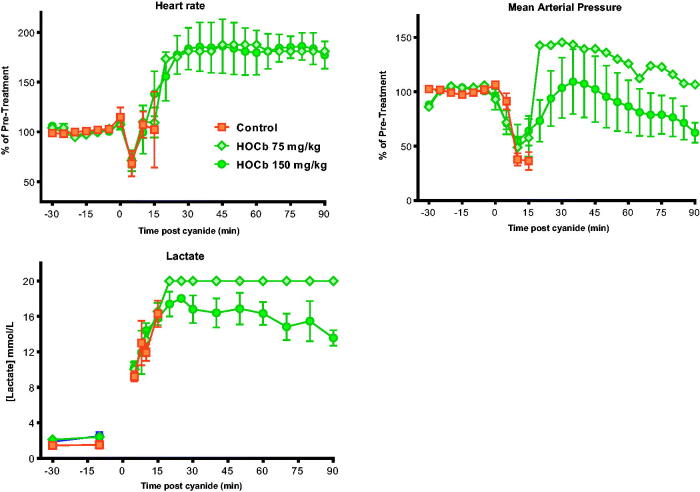
Effect of high dose hydroxocobalamin. Cardiovascular (heart rate, MAP) and metabolic (arterial lactate) effects of 0.15 mmol/kg KCN alone (square) or after pre-treatment with hydroxocobalamin 75 mg/kg (diamond) or 150 mg/kg (circle). Values are means ± SEM (for cardiovascular variables, of normalised responses [mean of -30 min and -10 min pre-cyanide exposure values = 100%]).

**Figure 6.**
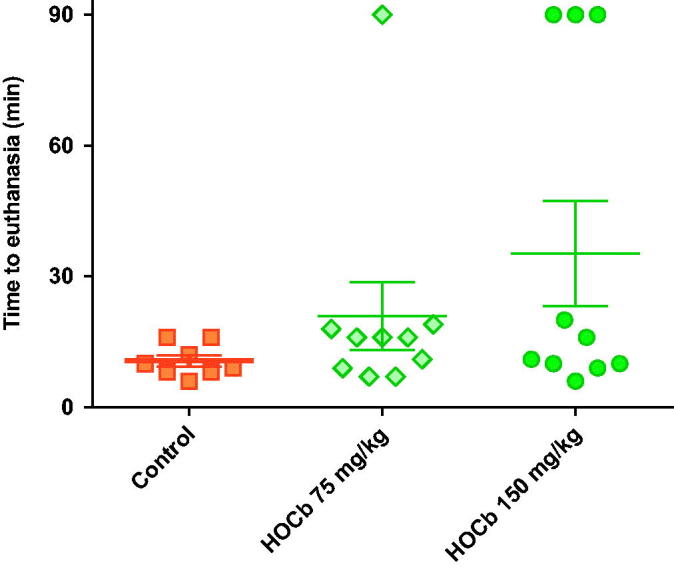
Survival after high dose hydroxocobalamin. Time to euthanasia after 0.15 mmol/kg KCN alone (square) or after pre-treatment with hydroxocobalamin 75 mg/kg (diamond) or 150 mg/kg (circle). The study had a planned maximum duration of 90 min. All animals undergoing euthanasia at this time had good cardiovascular function and were judged to have survived the procedure. Mean (SEM) time to euthanasia are shown for each group.

Control animals showed severe toxicity cardiovascular and metabolic toxicity (Figure 5) after 0.15 mmol/kg cyanide with euthanasia being required at a mean of 10 min post-exposure in all eight animals (Figure 6). There was a modest dose response in efficacy, with 75 mg/kg causing 1/10 animals to survive to 90 min, whilst 150 mg/kg caused 3/10 animals to survive to 90 min. There were no differences in the time to euthanasia in animals requiring this intervention (control: 10.6 (SEM: 1.3) min, 75 mg/kg arm: 13.2 (1.6) min, and 150 mg/kg arm: 11.7 (1.8) min). Surviving animals were hypotensive and tachycardic throughout the study.

**Figure 7.**
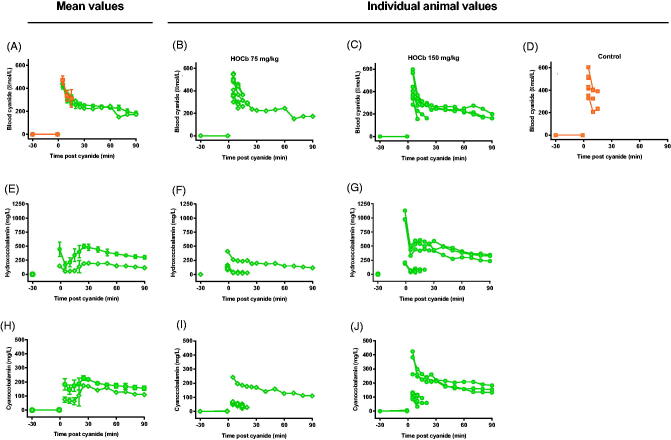
Pharmacokinetics of cyanide, hydroxocobalamin, and cyanocobalamin. Mean (SEM; A, E, H) and individual animal (B–D, F and G, I and J) concentrations of blood cyanide (µmol/L CN^−^, A–D), plasma hydroxocobalamin (mg/L, E–G), and plasma cyanocobalamin (mg/L, H–J) after administration of 75 (B, E, I) or 150 (C, F, J) mg/kg hydroxocobalamin or (D) plasma placebo.

Measurement of blood cyanide and hydroxocobalamin concentrations provided an explanation for the variation. Mean peak blood cyanide concentrations were similar at 5 min: control: 470 (SEM: 37) µmol/L; 75 mg/kg arm: 437 (29) µmol/L; and 150 mg/kg arm: 430 (35) µmol/L (Figure 7(A,D)). However, plasma hydroxocobalamin (Figure 7(B,E)) and cyanocobalamin (Figure 7(C,F)) concentrations showed markedly variable pharmacokinetics at both doses (hydroxocobalamin maximum 5.0 and 10.7-fold variation, and cyanocobalamin maximum 5.1 and 7.5-fold variation, for 75 mg/kg and 150 mg/kg doses, respectively). Animals surviving the cyanide challenge had substantially higher plasma hydroxocobalamin and cyanocobalamin concentrations compared to the other animals. There was a clear correlation between antidote effectiveness and plasma cobalamin concentrations (Figure 7).

## Discussion

The use of chemical weapons in attacks on civilians has increased markedly over the last decade, but there has been little progress in the clinical development and use of novel highly effective antidotes for treatment. The use of hydroxocobalamin has been heavily promoted for treatment of cyanide toxicity [6,8,9,34–36] but there is little comparative evidence on efficacy. In this study of minipigs poisoned with a rapid intravenous infusion of potassium cyanide, although both hydroxocobalamin and dicobalt edetate showed some efficacy at minimally toxic doses, neither were highly effective. Hydroxocobalamin – when given in double the licenced dose and at eight times the recommended infusion rate (equivalent human dose: 10 g over 10 min) – was effective for some animals at but not others. Its efficacy was limited by the highly variable pharmacokinetics that have been previously noted.

Even this modest benefit is unlikely to occur in chemical weapon attacks, when there is rapid inhalation of cyanide gas. We gave the antidotes shortly before the cyanide to maximise the chance of benefit. However, ventilated animals that did not receive an antidote required euthanasia due to cardiotoxicity within ten minutes of administration of a relatively modest dose of cyanide (substantially less than 2× the LD50). Unless the antidotes can be given before or within a few minutes of exposure, as done by the military with oximes and atropine for OP nerve agent attacks [37], it seems unlikely that they will save many lives. It might be possible to infuse hydroxocobalamin as additional protection into first responders entering a scene with cyanide released but, even in this prophylactic situation, protection would be modest.

Arterial lactate is used as a marker of high toxicity, with concentrations greater than 10 mmol/L associated with moderate-severe toxicity [4]. However, in our model, the usually sub-lethal 0.08 mmol/kg (5.25 mg/kg) dose produced similar lactate rises to the lethal 0.12 mmol/kg (7.5 mg/kg) dose. In addition, pre-treatment with effective doses of antidote at the 0.12 mmol/kg dose did not alter the rise in lactate concentration, suggesting that high arterial lactate concentrations may not be a reliable biomarker of cyanide poisoning and severity.

Hydroxocobalamin was associated with no apparent adverse effects, while the double dose of dicobalt edetate caused marked hypertension that settled over 10–20 min. This adverse effect has been noted previously [10]. A drug that causes hypertension might be beneficial to patients with severe cyanide poisoning in whom hypotension is expected. However, we saw no such beneficial effect in this model. There are few data on dicobalt edetate pharmacokinetics in humans or pigs. It likely distributes rapidly into the extracellular fluid where it binds cyanide. Since it has a long elimination half-life and was given just before the cyanide dose, it is unlikely that rapid clearance was responsible for its poor efficacy.

The modest efficacy of hydroxocobalamin was unexpected. The half-life of hydroxocobalamin following IV or intraosseous administration in minipigs is 145 min [13]. Therefore clearance is not responsible for its lack of efficacy. Instead, it is more likely to be related to limited cyanide binding capacity (Box 1) and poor ability to reduce circulating free cyanide concentrations to sub-toxic levels.

The difference with previous studies of hydroxocobalamin that have reported efficacy may be due in part to the different peak blood cyanide concentrations in the models. Peak concentrations in this ventilated model (685 μM [17.8 mcg/mL] for a dose of 0.15 mmol/kg NaCN) were substantially higher than reported from others models, ranging for example from 64.5 μM (1.68 mg/L) [22] to 130.6 μM (3.4 mg/L) [38]. However, the pig results are consistent with blood concentrations measured after lethal cyanide poisoning in humans (150–1700 μM) [39]. Pigs with a mean blood concentration of 329 µmol/L, after 0.08 mmol/kg, survived without antidotes. Of note, animals euthanised after 0.12 mmol/kg had only slightly higher blood cyanide concentrations; however, the small numbers preclude detailed assessment of plasma CN concentrations associated with mortality.

The dose of cyanide found to be survivable in this study (0.08 mmol/kg, 5.25 mg/kg) was higher than uniformly fatal doses in other studies eg. 4.91 (SD: 1.3) mg/kg in the study of Bebarta et al. [23]. The duration of dose administration (40 s vs. 25 min) in the two studies is likely to be the cause for the difference in the lethal dose (as well as in time to death and lactate concentration), perhaps with a contribution from differences in pig strain (in-bred Gottingen vs. out-bred Yorkshire). The approximate lethal dose in our studies of 400–500 mg for a 75 kg adult was about 2-fold higher than the literature’s estimate of 200–300 mg, likely due to differences between the model and human poisoning.

Peak blood cyanide concentration in the low dose group which survived was 328 mol/L (8.5 mg/L). This is significantly higher than published human concentrations, which suggest that concentrations >3 mg/L are lethal [40]. However, the peak concentration was measured only five min after rapid dosing. In clinical practice, blood is rarely taken for cyanide measurement so soon after exposure, nor is it (usually) taken from a live patient as occurred with these animals. The blood concentrations in this study were therefore expected to be significantly higher than those measured in clinical cases, many of which were post-mortem.

Hydroxocobalamin was registered with the European Medicines Agency on the basis of a clinical dossier comprising one safety study and four non-comparative efficacy studies (one prospective, three retrospective) [12]. None of these human studies can confirm effectiveness. To overcome this lack of data, a placebo-controlled canine study was performed. This study involved infusing KCN until the dogs had been apnoeic for three minutes (mean total dose: 2.2–2.4 mg/kg), whereupon the cyanide was stopped and animals administered placebo, or hydroxocobalamin 75 or 150 mg/kg [17]. In this “precipice” model (see introduction), both doses of hydroxocobalamin were effective.

We initially tested the UK’s licenced adult dose of hydroxocobalamin 5 g (71.4 mg/kg in a 70 kg adult), 0.054 mmol/kg assuming a molecular weight of 1382 for hydroxocobalamin HCl. If this total amount is available to react with CN^−^ soon after administration, an equivalent molar quantity of CN^−^ could be bound (Box 1). These simple calculations suggest that, at best, the licenced dose of hydroxocobalamin can bind around half a minimum lethal dose (about 0.06 mmol/kg of KCN) and the double dose should be able to bind a just lethal dose.

Although there are some concerns about the effectiveness of hydroxocobalamin for smoke cyanide toxicity [41], its administration to self-ventilating patients extricated from house fires with presumed cyanide toxicity is widely recommended [6,8,42,43]. Our study suggests that patients with cyanide exposure as indicated by high lactate concentrations who have stable cardio-vascular function may survive without antidotes, and that antidotes only offer limited additional protection. In the absence of controlled clinical trials, the available evidence does not support routine administration of the cyanide antidotes to unconscious patients exposed to house fires [41]. A cluster RCT comparing the administration of hydroxocobalamin by ambulance paramedics to unconscious patients at the scene of the exposure with an equivalent amount of 0.9% sodium chloride solution would provide the data required to determine whether this approach is effective.

### Limitations

The study was carried out in pigs because well controlled human studies are not possible. However, porcine cardiorespiratory physiology is similar to humans [20] and human equivalent doses of antidotes were given. We performed an initially small focussed study that was not powered to distinguish small differences in time to death following treatment with different antidotes. We finally compared hydroxocobalamin in a larger study that was powered to detect substantial differences in mortality, but this did not show reliable efficacy.

In order to optimise cardiovascular stability and reduce both variability and number of animals required, the study was performed in anaesthetized pigs in which tracheal intubation and positive pressure lung ventilation were imposed. The time to apnoea was therefore not recorded. This does not match the real-life situation in which patients would not be intubated or ventilated. An ability to prevent respiratory arrest in a self-ventilating patient at lower cyanide doses might increase the benefit from cyanide antidotes. However, this would again require early treatment.

Due to a local lack of inhalational facilities to perform hydrogen cyanide studies in anaesthetized and ventilated pigs, we chose to give bolus doses of KCN to simulate sudden massive cyanide inhalation. This situation differs from that following ingestion of cyanide salts in which death would be expected to occur later.

The study also looked at mortality but not long-term sequelae; it is possible (but perhaps unlikely) that both antidotes are highly effective at reducing such sequelae while only moderately reducing death.

## Conclusion

In these porcine studies, we found modest beneficial effects from double doses of hydroxocobalamin or dicobalt edetate given immediately before a just lethal exposure to cyanide. Increased doses may improve effectiveness but have not been tested either pre-clinically or clinically. More effective antidotes are urgently required. Candidates include cobinamide, an analogue of hydroxocobalamin with twice the molar cyanide binding capacity [15,22], and sulfanegen, a dimeric prodrug of 3-mercaptopyruvate that is a substrate for the cyanide-detoxifying enzyme 3-mercaptopyruvate sulfurtransferase [15,24]. Recently, a liposomal construct containing rhodanase and a novel sulphur donor has been reported to increase the LD_50_ of KCN 15-fold when given prophylactically to mice [44], a far higher level of protection than given by the antidotes discussed here.

## Supplementary Material

Supplemental Material
