# Supplementary material for: Modest and variable efficacy of pre-exposure hydroxocobalamin and dicobalt edetate in a porcine model of acute cyanide salt poisoning
Source: Clin Toxicol (Phila). 2019 Aug 7;58(3):190–200. doi: 10.1080/15563650.2019.1628969 (PMC7034532; doi:10.1080/15563650.2019.1628969)
Supplement: Supplemental Material [file ICTX_A_1628969_SM4431.docx]

**Online supplement for methods**

***1. Hydroxocobalamin and Cyanocobalamin***

Plasma and whole blood hydroxocobalamin and cyanocobalamin were analysed at the Medical Toxicology Centre, Newcastle University by LC-MS-MS, using a method based on the work of Schwertner et al [1] and Chaissaigne et al [2]. All solvents, acids and buffers were of HPLC grade and obtained from Fisher Scientific, Loughborough, UK, unless otherwise stated.

**Stock Solutions**

Hydroxocobalamin hydrochloride (Sigma-Aldrich, St. Louis, MO, USA) was weighed into an amber vial and sufficient deionised water added to achieve a stock concentration of 10mg/mL. Stock solutions of cyanocobalamin (Sigma-Aldrich, St. Louis, MO, USA) were prepared in a similar manner. These stock standard solutions were stored at 4°C and then further diluted in deionised water to produce mixed standards immediately prior to use.

**Sample Preparation**

Minipig plasma (10 µL) was mixed with deionised water (90 µL). Aqueous 1% (v/v) acetic acid (200 µL) was then added to the diluted sample, which was then vortex mixed. These acidified and diluted minipig plasma samples were then subjected to SPE (Hypersep C18, 100mg, Thermo Scientific, Bellefonte, PA, USA) in a 96-well plate format as follows: The SPE phase was initially conditioned with methanol (500 µL), followed by aqueous 1% (v/v) acetic acid (500 µL). Acidified plasma samples were then loaded onto the SPE plate, which was then washed with aqueous 1% acetic acid (500 µL). The cobalamins were then eluted from the SPE plate with methanol (2 mL), which was then removed under a stream of nitrogen, at 50°C (TurboVap, Caliper Life Sciences Inc., Hopkinton, MA, USA). Dried down extracts were reconstituted into 1mL of 90:10 water:methanol + 0.1% formic acid. Calibration standards were matrix matched using blank (i.e. pre-exposure) minipig plasma samples acquired during the course of the animal studies.

Hydroxocobalamin and cyanocobalamin were analysed by positive mode liquid chromatography electrospray ionisation mass spectrometry - LC-ESI-MS-MS - using a Shimadzu Prominence XP UPLC system coupled to a Quantum Ultra triple quadrupole mass spectrometer, controlled using Xcalibur 2.1 software (Thermo). Separation of the cobalamins was performed on a reversed-phase HPLC column (Waters XBridge, C18, 2.5µm, 50mm x 2.1mm) using a gradient of 90:10 water:methanol + 0.1% formic acid to 5:95 water:methanol + 0.1% formic acid, over 15 minutes, at a flow rate of 200 µL/min. The injection volume was 5 µL, and the column was maintained at a temperature of 40°C. MRM transitions monitored were 665.1→146.9 and 665.1→912.6 for hydroxocobalamin and 678.3→146.9 and 678.3→912.6 for cyanocobalamin. Chromatographic peaks were integrated using XCalibur 2.1 software. External standard calibration curves were constructed using matrix matched standards. Extraction efficiencies were monitored using spiked plasma or whole blood. The calculated values for these standards were between 85 to 103% of the target value.

***2. Cyanide***

A LC-ESI-MS-MS method to measure cyanide in whole blood was based on the work of Tracqui [3] and Lacroix [4]. Cyanide was derivatised by reaction with 2,3-naphthalene dicarboxaldehyde (NDA) and taurine to form cyano[f]benzoisoindole (CBI) derivatives to render it amenable to analysis by LC-ESI-MS-MS. All solvents, acids and buffers were of LC-MS grade and obtained from Fisher Scientific, unless otherwise stated.

**Stock Solutions**

Potassium cyanide (Fluka) stock standard solutions (~500 µg/mL, as CN) were prepared in aqueous 0.1 M NaOH. The CN stock solution was diluted 100-fold in 0.1 M NaOH to generate working cyanide standard solutions (~5 µg/mL), which were used to spike blank blood samples to generate matrix matched calibration standards. Labelled ^13^C^15^N internal standard stock solutions (~5000 µg/mL) were also prepared in 0.1 M NaOH from the K^13^C^15^N salt (Isotec). Two working standard solutions of ^13^C^15^N (4 µg/mL and 40 µg/mL) in 0.1 M NaOH were prepared from the ^13^C^15^N stock solution. Cyanide stock solutions were stored at 4°C and then further diluted to create the working standards described above. Solutions of the cyanide derivatisation reagents NDA (Fluka) and taurine (Sigma) were prepared immediately prior to use in amber vials, with the aid of sonication. Taurine solutions (~5 mM) were prepared in 100 mM/pH~8.5 ammonium bicarbonate/carbonate buffer, and solutions of NDA (~5 mM) were prepared in methanol.

**Sample Preparation**

Minipig whole blood samples (25 µL) were spiked with internal standard solution (10 µL of either 4 µg/mL or 40 µg/mL ^13^C^15^N, dependant on anticipated cyanide level in the sample) and 0.1 M NaOH (20 µL). In the case of matrix matched standards, the 0.1 M NaOH solution was omitted, and replaced with a suitable volume of ~5 µg/mL cyanide standard, plus sufficient 0.1 M NaOH to be equivalent to the total volume of 0.1 M NaOH that was added to the samples. Cyanide calibration standards were matrix matched using blank (i.e. pre-exposure) minipig whole blood samples acquired during the course of the animal studies. Following sample spiking, 100 mM, pH~8.5 ammonium bicarbonate/carbonate buffer (200 µL) was added to each sample. After thorough mixing, methanol (700 µL) was added to the diluted blood samples. Samples were centrifuged (16,100 x g, at room temperature for 5 minutes) in a microfuge (Eppendorf), following further mixing. An aliquot (250 µL) of the supernatant was removed from the centrifuge tube and then placed in a glass tube containing 5 mM NDA solution (10 µL) and 5 mM taurine solution (50 µL) to form the CBI derivatives. Reaction mixtures were incubated at room temperature in darkness for ~1 hour, then taken down to dryness under a stream of nitrogen (TurboVap) and reconstituted in 10 mM/pH~8.5 ammonium bicarbonate/carbonate buffer containing 10% methanol (1 mL). For samples that had been spiked with the 40 µg/mL ^13^C^15^N internal standard it was necessary to dilute the reconstituted extract a further 10-fold in 10 mM/pH~8.5 ammonium bicarbonate/carbonate buffer containing 10% methanol prior to ESI LC –MS-MS analysis.

**Cyanide ESI LC –MS-MS Analysis**

Cyanide CBI derivatives were analysed by negative mode ESI LC-MS-MS, using a Shimadzu Prominence LC system coupled to an AB Sciex QTRAP 5500 mass spectrometer, controlled using Analyst 1.5.1 software (AB Sciex). The QTRAP 5500 was operated in Multiple Reaction Monitoring (MRM) mode and was equipped with a Turbo V ion source and ESI probe. The MRM transitions acquired were; m/z 298.9 → m/z 191.0, m/z 300.8 → m/z 193.0 and m/z 298.9 → m/z 80.9.

A mobile phase stock solution of 100 mM, pH8.5 ammonium bicarbonate/carbonate buffer was prepared in water and then diluted 10-fold in either water (mobile phase A), or in methanol (mobile phase B). Separation of the CBI cyanide derivatives was performed on a reversed-phase HPLC column (Waters XBridge, C18, 2.5µm, 50mm x 2.1mm) equipped with a guard cartridge of the same material. The mobile phase flow rate was 200 µL/min, injection volume was 5 µL, and the column was maintained at a temperature of 40°C. The LC Gradient elution programme was as follows: linear gradient from 10% B to 95% B over 10 minutes, held at 95% B for 5 minutes, and finally returned to initial conditions of 90% A and 10% B for 5 minutes to allow column re-equilibration.

Chromatographic peaks for CN-CBI (m/z 298.9 → m/z 191.0 transition) and ^13^C^15^N-CBI (m/z 300.8 → m/z 193.0 transition) derivatives were integrated using the MultiQuant 2.1.1 software (AB Sciex). Internal standard calibration curves were constructed using the peak areas from the above MRM transitions for the quantification of minipig blood cyanide concentrations.

**REFERENCES**

1. Schwertner HA, Valtier S, Bebarta VS. Liquid chromatographic mass spectrometric (LC/MS/MS) determination of plasma hydroxocobalamin and cyanocobalamin concentrations after hydroxocobalamin antidote treatment for cyanide poisoning. J Chromatogr B Analyt Technol Biomed Life Sci. 2012;905:10-16.

2. Chaissaigne H, Lobinski R. Determination of cobalamins and cobinamides by microbore reversed-phase HPLC with spectrophotometric, ion-spray ionization MS and inductively coupled plasma MS detection. Analytica Chim Acta. 1998;359:227-235.

3. Tracqui A, Raul JS, Geraut A, et al. Determination of blood cyanide by HPLC-MS. J Anal Toxicol. 2002;26:144-148.

4. Lacroix C, Saussereau E, Boulanger F, et al. Online liquid chromatography-tandem mass spectrometry cyanide determination in blood. J Anal Toxicol. 2011;35:143-147.
